# Enhancing precision in cancer treatment: the role of gene therapy and immune modulation in oncology

**DOI:** 10.3389/fmed.2024.1527600

**Published:** 2025-01-13

**Authors:** Emile Youssef, Brandon Fletcher, Dannelle Palmer

**Affiliations:** Kapadi, Inc., Raleigh, NC, United States

**Keywords:** gene therapy, immuno-oncology, CAR-T cell therapy, CRISPR-Cas9, gene editing, tumor microenvironment (TME), ferroptosis, combination therapies

## Abstract

Gene therapy has long been a cornerstone in the treatment of rare diseases and genetic disorders, offering targeted solutions to conditions once considered untreatable. As the field advances, its transformative potential is now expanding into oncology, where personalized therapies address the genetic and immune-related complexities of cancer. This review highlights innovative therapeutic strategies, including gene replacement, gene silencing, oncolytic virotherapy, CAR-T cell therapy, and CRISPR-Cas9 gene editing, with a focus on their application in both hematologic malignancies and solid tumors. CRISPR-Cas9, a revolutionary tool in precision medicine, enables precise editing of cancer-driving mutations, enhancing immune responses and disrupting tumor growth mechanisms. Additionally, emerging approaches target ferroptosis—a regulated, iron-dependent form of cell death—offering new possibilities for selectively inducing tumor cell death in resistant cancers. Despite significant breakthroughs, challenges such as tumor heterogeneity, immune evasion, and the immunosuppressive tumor microenvironment (TME) remain. To overcome these barriers, novel approaches like dual-targeting, armored CAR-T cells, and combination therapies with immune checkpoint inhibitors and ferroptosis inducers are being explored. Additionally, the rise of allogeneic “off-the-shelf” CAR-T therapies offers scalable and more accessible treatment options. The regulatory landscape is evolving to accommodate these advancements, with frameworks like RMAT (Regenerative Medicine Advanced Therapy) in the U.S. and ATMP (Advanced Therapy Medicinal Products) in Europe fast-tracking the approval of gene therapies. However, ethical considerations surrounding CRISPR-based gene editing—such as off-target effects, germline editing, and ensuring equitable access—remain at the forefront, requiring ongoing ethical oversight. Advances in non-viral delivery systems, such as lipid nanoparticles (LNPs) and exosomes, are improving the safety and efficacy of gene therapies. By integrating these innovations with combination therapies and addressing regulatory and ethical concerns, gene therapy is poised to revolutionize cancer treatment, providing durable, effective, and personalized solutions for both hematologic and solid tumors.

## 1 Introduction

The concept of gene therapy dates back to the 1960s, but its clinical application became a reality in the 1990s. A pivotal moment occurred in 1990 when Dr. W. French Anderson conducted the first approved gene therapy trial, treating a young patient with adenosine deaminase deficiency using retroviral vectors to introduce a functional ADA gene into the patient’s T-cells (1). This breakthrough illustrated the potential for gene therapy to treat genetic diseases at their root cause, laying the foundation for future therapeutic developments.

Although this early success sparked widespread enthusiasm, the field faced significant setbacks in the late 1990s. Most notably, the tragic death of Jesse Gelsinger during a clinical trial for ornithine transcarbamylase deficiency in 1999 raised serious safety concerns and led to increased regulatory scrutiny (2). Nevertheless, gene therapy rebounded through technological advancements in vector safety and gene transfer efficiency during the early 2000s.

Building upon early successes and challenges, gene therapy has since evolved, driven by advancements in vector safety and gene transfer efficiency, positioning it as a cornerstone in modern oncology. Gene therapy is revolutionizing the field of oncology by offering precise, targeted treatments aimed at the underlying genetic causes of cancer. These therapies have emerged as a crucial component of modern cancer treatment, addressing the limitations of conventional treatment approaches like chemotherapy and radiation. Gene therapies enable personalized interventions, ranging from the correction of faulty genes to the enhancement of immune responses against tumors, thereby offering new avenues for treating both hematologic and solid malignancies (3, 4). In 2003, China approved Gendicine, the world’s first gene therapy for cancer, which delivers the p53 tumor suppressor gene to treat head and neck squamous cell carcinoma (5).

One of the most significant breakthroughs in gene therapy came with the advent of Chimeric Antigen Receptor T-cell (CAR-T) therapy, which modifies a patient’s T-cells to express receptors that specifically target cancer antigens. Although not approved as a first-line therapy, CAR T-cell therapy has transformed the treatment landscape for certain aggressive, relapsed, or refractory lymphomas and multiple myeloma.

Simultaneously, the discovery of the Clustered Regularly Interspaced Short Palindromic Repeats-Associated Protein 9 (CRISPR-Cas9) gene-editing system in 2012 represented a new era for gene therapy, allowing precise, targeted modifications to DNA sequences. CRISPR-Cas9 has since become a key tool in the development of gene therapies, enabling scientists to edit or disrupt specific genes with greater accuracy than previous technologies (6). By 2019, CRISPR-based therapies entered human clinical trials, further advancing the use of gene editing in cancer and other genetic disorders (7).

Today, gene therapy continues to advance with innovative approaches such as oncolytic virotherapy, which uses viruses to kill cancer cells selectively, and allogeneic CAR-T therapies, which provide “off-the-shelf” treatments using donor T-cells (8, 9). Together with advancements in gene-editing technologies like base editing and prime editing, these innovations hold the potential to overcome the limitations of current cancer treatments and expand the scope of gene therapy across oncology (10).

Decades of scientific breakthroughs and regulatory and clinical successes have positioned gene therapy as a cornerstone of modern cancer treatment (11). It offers hope for more effective, durable, and personalized cancer therapies in the future. Gene therapies have offered transformative solutions for patients with previously untreatable conditions. As Peter Marks, MD, PhD, Director of the Food and Drug Administration (FDA)‘s CBER, emphasized at the 2023 Cell & Gene Meeting on the Mesa: ‘We may not believe in miracles. But there are miraculous, and this is one.’ This highlights the regulatory focus on ensuring the safe development of gene therapies for small populations, with the goal of expanding access to broader groups in the future.

The landscape of gene therapy in oncology continues to evolve rapidly, driven by unprecedented advancements in precision medicine, gene-editing technologies, and innovative therapeutic strategies. With the increasing integration of immuno-oncology, gene therapies such as CAR-T, CRISPR-Cas9, and oncolytic virotherapy are expanding their potential in both hematologic and solid tumors. These therapies offer highly personalized, targeted treatments that not only address the genetic drivers of cancer but also aim to enhance immune responses, providing hope for more durable and effective solutions in cancer care. However, challenges such as tumor heterogeneity, immune evasion, and the complexities of the tumor microenvironment (TME) must be overcome to unlock the full potential of gene therapy. As the field continues to progress, the collaboration between gene therapy and immune modulation strategies represents one of the most promising paths forward, laying the foundation for the next generation of cancer therapies. This review will explore the current landscape of gene therapies in oncology, addressing both their groundbreaking potential and the challenges that must be surmounted to bring these therapies into mainstream clinical practice.

## 2 Types of gene therapy modalities in oncology

Gene therapies in oncology offer several approaches to targeting cancer, each with distinct mechanisms of action. Recent advancements have expanded the scope and effectiveness of these therapies, improving patient outcomes by addressing critical challenges like precision, targeting, and delivery (Table 1).

**Table 1:** Gene therapy modalities in oncology.

Therapy type	Mechanism of action	Applications	Challenges	References
Gene replacement	Introduces functional copies of defective genes	Solid tumors (e.g., breast, lung cancer)	Viral vector safety, off-target effects	(6, 12)
Gene silencing	Inhibits oncogene expression using RNAi	Pancreatic and liver cancers	Delivery, off-target silencing	(13, 17, 22)
Suicide gene therapy	Converts prodrugs into cytotoxic agents	Gliomas, pancreatic cancer	Delivery precision, enhancing bystander effect	(30, 32, 33)
Oncolytic virotherapy	Uses viruses to selectively lyse cancer cells	Melanoma, prostate, and pancreatic cancers	Immune response, delivery efficiency	(24, 28, 29)
CAR-T cell therapy	Modifies T-cells to target specific antigens	Hematologic malignancies	Limited efficacy in solid tumors	(38, 40, 42)

### 2.1 Gene replacement therapy

Gene replacement therapy introduces functional copies of defective or missing genes into cancer cells to restore normal cellular function. This therapy is particularly beneficial for cancers driven by specific genetic mutations, such as Tumor Protein 53 (TP53). Among the various viral delivery systems discussed later, Gendicine, an adenoviral vector (AdV) delivering the p53 gene, has been successfully employed in the treatment of head and neck squamous cell carcinoma. This achievement represents a notable milestone in advancing gene therapy for cancer (12). Recent advancements in adeno-associated viral (AAV) vectors have further improved the safety and targeting precision of gene replacement therapies, making them viable options for a wider range of oncological applications, including solid tumors like breast, lung, and colon cancers (6). These therapies utilize viral vectors such as AAV to deliver therapeutic genes directly to the tumor site, with the potential for durable responses and, in some cases, functional cures.

Moreover, the rise of CRISPR/Cas9 technology offers an additional layer of precision, enabling not only gene addition but also direct correction of mutations that drive tumorigenesis. Unlike traditional gene replacement methods that insert a functional gene copy, CRISPR-Cas9 allows for the *in situ* correction of genetic defects, including those involved in dominant-negative mutations, making it especially useful for cancers like colorectal carcinoma (4). However, while CRISPR presents an exciting opportunity for permanent correction of oncogenic mutations, challenges such as ethical concerns, off-target effects, and long-term safety still need to be addressed as discussed later.

### 2.2 Gene silencing therapy

Gene silencing therapy inhibits the expression of oncogenes that drive cancer progression, making it a critical approach in cancer treatment. RNA interference (RNAi) has emerged as a versatile and effective therapeutic strategy, with recent advancements in chemical modifications and delivery systems leading to the clinical approval of multiple siRNA-based drugs (13). These developments have improved the stability and efficacy of RNAi therapies while minimizing off-target effects (13, 14). RNAi, through small interfering RNAs (siRNA) or short hairpin RNAs (shRNA), target specific mRNAs for degradation, thereby preventing the production of dysfunctional proteins (15). For instance, targeting PLK1 via siRNA is being explored in clinical trials for pancreatic and liver cancers (16). Promising results from preclinical models of aggressive solid tumors further highlight the potential of RNAi-based therapies (17, 18). Additionally, gene silencing therapies using siRNA and miRNA have shown great potential in downregulating oncogenes that drive tumor progression (19, 20). siRNA targets specific mRNA sequences, preventing the production of proteins that fuel cancer growth. This approach has been applied in several cancers to silence genes involved in the aggressive types of cancer progression (2, 21). Furthermore, recent advances in the clinical translation of RNAi-based therapeutics have shown promise in downregulating oncogenes, offering a complementary approach to existing gene-editing technologies like CRISPR (22). miRNA therapy is also advancing in colon and lung cancers, playing a critical role in suppressing oncogenes and enhancing tumor suppression mechanisms (5, 23).

### 2.3 Oncolytic virotherapy

Oncolytic virotherapy uses genetically engineered viruses to infect and kill cancer cells selectively. The FDA-approved oncolytic virus Talimogene laherparepvec (Imlygic) has shown efficacy in treating melanoma (24). By infecting and lysing cancer cells, these viruses also stimulate an immune response, helping to attack remaining tumor cells (25). Researchers are now exploring oncolytic viruses in solid tumors like prostate and pancreatic cancers and enhancing the virus’s ability to penetrate tumors and amplify the immune response (26, 27). Recent studies, including Zou et al., (28) have demonstrated that combining oncolytic virotherapy with immune checkpoint inhibitors significantly amplifies the immune response against tumors, particularly in solid tumors where immune evasion mechanisms and the TME present significant challenges (28, 29).

### 2.4 Suicide gene therapy

Suicide gene therapy involves introducing genes into cancer cells that convert non-toxic prodrugs into cytotoxic agents, selectively inducing cell death within tumor tissues. One of the most well-established approaches utilizes the Herpes Simplex Virus Thymidine Kinase (HSV-TK) gene, which phosphorylates the antiviral drug ganciclovir, converting it into a toxic compound that kills dividing tumor cells (30). This strategy has shown promise in clinical trials, particularly for hard-to-treat cancers such as gliomas and pancreatic cancer (31, 32). In gliomas, the HSV-TK/ganciclovir system has been effective in reducing tumor volumes, primarily due to the bystander effect, where not only the genetically modified cells are killed, but also adjacent tumor cells. This effect occurs as cytotoxic metabolites diffuse from treated to neighboring cells, amplifying the therapeutic outcome (31, 32). This has led to promising results, especially when combined with conventional treatments like radiotherapy, which further sensitizes tumor cells to the cytotoxic effects of the prodrug (33).

Additionally, pancreatic cancer, a notoriously resistant solid tumor, has been a target of suicide gene therapy. Although early results have been encouraging, there is ongoing research to improve delivery methods and enhance the therapeutic effect. For instance, new delivery techniques using mesenchymal stem cells (MSCs) as carriers have been explored in preclinical studies (34, 35). These cells naturally home to tumor sites, improving the precision of suicide gene delivery and potentially enhancing therapeutic outcomes (36). MSCs can deliver the HSV-TK gene directly to the TME, ensuring localized activation of the prodrug and minimizing off-target effects However, challenges remain in increasing the efficacy of suicide gene therapy, especially in overcoming tumor resistance mechanisms, which limit the treatment’s success in aggressive cancers like pancreatic cancer. Additionally, while the bystander effect holds great potential for expanding the reach of the therapy beyond transduced cells, optimizing this effect remains a focus of ongoing research. Strategies to enhance the spread of cytotoxic metabolites and improve overall drug delivery are being actively investigated to increase the efficacy of HSV-TK-based therapies in broader tumor regions (37).

### 2.5 Autologous CAR-T cell therapy

**Figure 1:**
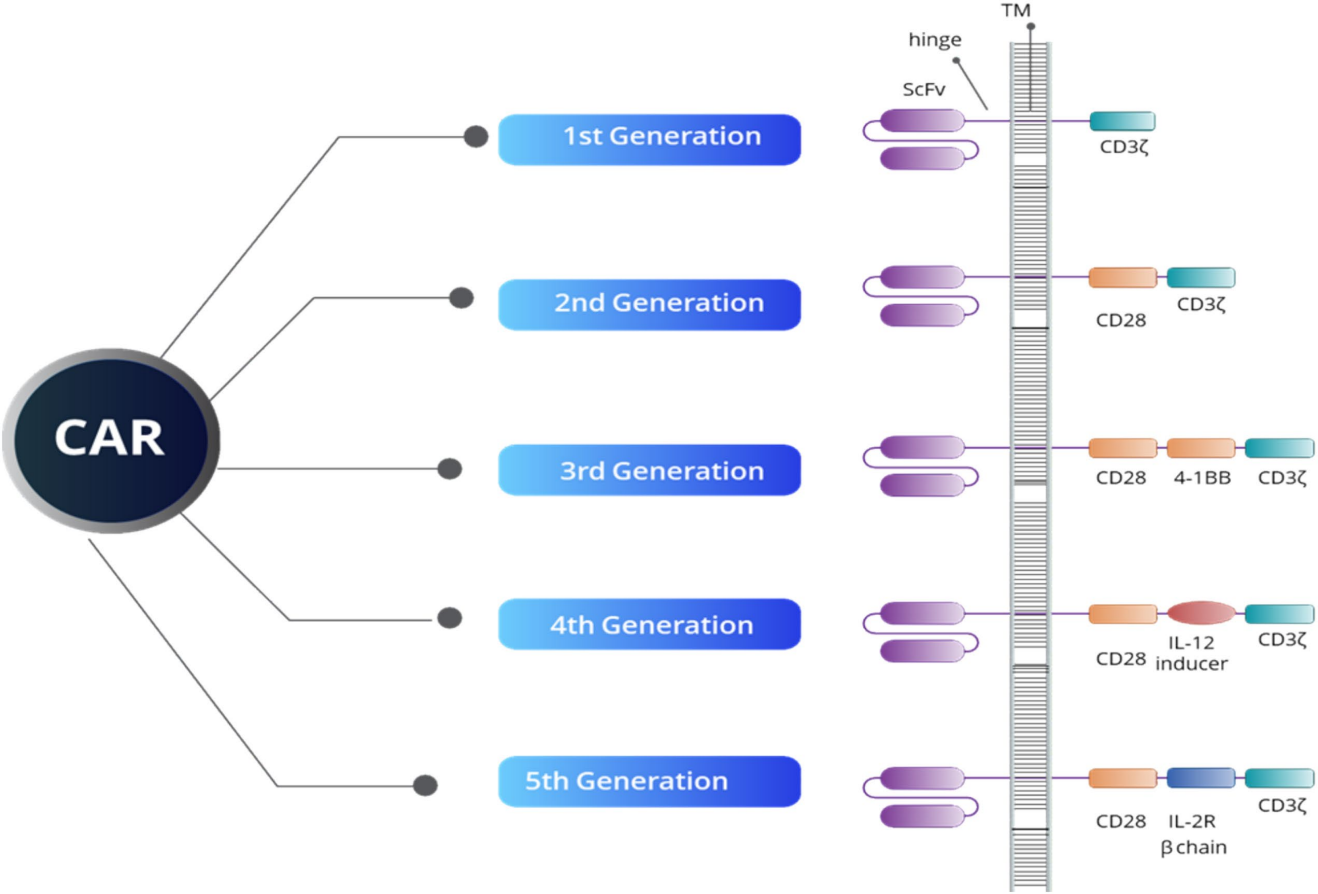
An overview of CAR structure reveals that all five generations of CAR constructs share four key domains: an extracellular domain that targets tumor-specific antigens (ScFV), a hinge region, a transmembrane domain (TM), and an intracellular domain. The intracellular domain’s structure defines both the generation of the CAR and its functional capacity. For example, the CD3ζ domain is crucial for initiating signal transduction pathways responsible for T-cell activation, proliferation, cytokine release, and cytotoxic activity. Additionally, the CD28 and 4-1BB domains serve as co-stimulatory signals, enhancing T-cell activation, longevity, and effectiveness. The IL-12 inducer domain facilitates cytokine production in the TME, while the IL-2R beta chain simulates IL-2 signaling, boosting CAR-T cell survival, proliferation, and persistence.

Since its inception, CAR-T therapy has undergone significant evolution, as illustrated in Figure 1, CAR-T therapy emerging to improve efficacy and safety. The first-generation CAR-T cells featured a basic design with a single signaling domain (CD3ζ), primarily for T cell activation. In the second generation, costimulatory domains such as CD28 or 4-1BB were added, improving T cell expansion and persistence. The third generation further enhanced T cell functionality by combining multiple costimulatory domains. Fourth-generation CAR-T, also known as TRUCKs (T cells redirected for antigen-unrestricted cytokine-initiated killing), introduced cytokine signaling, such as Interleukin-12 (IL-12), to strengthen the immune response within the TME. The fifth generation incorporated even more sophisticated signaling pathways to better target tumors and counteract immunosuppression induced by the TME (38). Autologous CAR-T cell therapy involves collecting a patient’s own T cells, genetically engineering them to express a chimeric antigen receptor (CAR) targeting specific tumor antigens, and re-infusing them into the patient. This highly personalized immunotherapy has revolutionized the treatment of hematologic malignancies, particularly for relapsed or refractory B-cell malignancies, such as leukemia and lymphoma (38, 39).

The FDA has approved several autologous CAR-T-cell therapies, including brexucabtagene autoleucel for adults with mantle cell lymphoma (MCL) and B-cell precursor acute lymphoblastic leukemia (ALL), tisagenlecleucel for pediatric and adult patients with relapsed or refractory ALL and large B-cell lymphoma, axicabtagene ciloleucel for relapsed or refractory large B-cell lymphoma and follicular lymphoma, and lisocabtagene maraleucel for relapsed or refractory large B-cell lymphoma. In multiple myeloma, Abecma (idecabtagene vicleucel) and Carvykti (ciltacabtagene autoleucel), both targeting B-cell maturation antigen (BCMA), have shown promise in treating relapsed or refractory cases, offering new options for patients who have exhausted conventional therapies (40–42). These therapies harness the patient’s immune system to attack and destroy cancer cells, but challenges remain, such as high manufacturing costs, logistical complexities, and limited efficacy in solid tumors (38, 39). Solid tumors, such as gliomas, present additional unique obstacles, further complicating the development of effective CAR-T therapies for these types of cancers due to their dense extracellular matrix and immunosuppressive microenvironment, which hinder the effective penetration and activity of CAR-T-cells. Recent studies suggest that optimizing CAR design could help overcome these obstacles. For instance, incorporating costimulatory domains such as 41BB and CD28 has enhanced CAR T-cell persistence and functionality *in vitro*. However, translating these improvements into *in vivo* settings remains a challenge (43, 44). A recent investigation into using B7-H3-specific CAR-T-cells for gliomas highlighted how CAR design can influence the recruitment and activation of immune cells, such as tumor-associated macrophages, within the brain TME (35). Such advancements suggest that further optimization of CAR-T cell therapies, mainly through engineering designs that modulate the immune microenvironment, may lead to improved outcomes in solid tumors like gliomas, where immune suppression and tumor heterogeneity continue to limit treatment efficacy (45).

### 2.6 Allogeneic CAR-T cell therapy

Although autologous CAR-T therapy has demonstrated significant efficacy in treating certain cancers, it also presents challenges, including lengthy manufacturing times, high costs, and patient-specific variability. These issues are especially pronounced in patients with extensive prior treatments or compromised immune systems. To address these limitations, allogeneic CAR-T therapy, often referred to as “off-the-shelf” therapy, has emerged as a promising alternative (9). This approach uses donor-derived T-cells that are genetically engineered to express CARs and modified to avoid immune rejection by the recipient’s body (46). One of the key benefits of allogeneic CAR-T therapy is its ability to pre-manufacture and store CAR-T cells, making them readily available for use in multiple patients. This approach not only addresses scalability issues but also enhances the accessibility of these treatments compared to the more individualized autologous method. Furthermore, allogeneic CAR-T cells provide an option for patients with compromised immune systems or insufficient healthy T-cells—those for whom autologous treatments may not be feasible due to prior treatments or other health factors (47, 48). Additionally, allogeneic CAR-T cells offer faster production times, which is critical for patients with aggressive cancers who cannot afford to wait for the lengthy manufacturing process of autologous therapies (49).

In terms of cost, allogeneic CAR-T therapies enable bulk manufacturing, which significantly lowers production expenses and makes these treatments more affordable and accessible to a wider patient population. As such, allogeneic CAR-T therapy represents an important step forward in overcoming the limitations of autologous treatments and expanding access to life-saving cancer therapies (9, 50). Several allogeneic CAR-T therapies have entered clinical trials, demonstrating promising results in hematologic malignancies:

- UCART19: Developed by Cellectis, UCART19 is an allogeneic CAR-T therapy targeting CD19, a common antigen in B-cell malignancies such as ALL and B-cell lymphoma. Early-phase trials in patients with relapsed or refractory ALL have shown that UCART19 can induce remission, offering hope to patients who have exhausted other treatment options (51). The CALM clinical trial, initiated in 2016, evaluated UCART19 as an ‘off-the-shelf’ CAR T-cell product in adult patients with relapsed or refractory B-cell acute lymphoblastic leukemia (B-ALL). Final results, published in 2023, demonstrated a manageable safety profile and promising antileukemic activity, with 48% of treated patients achieving complete remission lasting an average of 7.4 months. Notably, patients who received alemtuzumab as part of the lymphodepletion regimen showed higher levels of UCART19 expansion and an improved disease response (52).
- ALLO-501 and ALLO-715: Developed by Allogene Therapeutics, ALLO-501 is an allogeneic CAR-T therapy targeting CD19, currently under investigation in patients with relapsed or refractory non-Hodgkin lymphoma (NHL). In parallel, ALLO-715 targets BCMA in patients with multiple myeloma. Both therapies have shown promising early results regarding safety and efficacy, suggesting that off-the-shelf CAR-T therapies could play a crucial role in the future of cancer immunotherapy (53). The ALPHA and ALPHA2 Phase 1 trials assessed ALLO-501 and its next-generation variant, ALLO-501A, in patients with relapsed or refractory NHL. Updated data presented in 2023 demonstrated that ALLO-501A has a manageable safety profile, with no dose-limiting toxicities or graft-versus-host disease (GvHD). Efficacy outcomes were comparable to those of autologous CAR T-cell therapies, with an overall response rate of 75% and a complete response rate of 53% across various histologies in CAR T-cell naïve patients.
- PBCAR0191: Precision BioSciences has developed PBCAR0191, an allogeneic CAR-T therapy targeting CD19. Preliminary clinical trials for patients with B-cell acute lymphoblastic leukemia (B-ALL) and NHL show encouraging responses with manageable safety profiles (53).
- Precigen’s UltraCAR-T® is utilizing an innovative system for a non-viral, multigene delivery process that allows for rapid, decentralized manufacturing, enabling same-day engineering and next-day infusion of T cells into patients. UltraCAR-T® cells are designed to express a (CAR, membrane-bound interleukin-15) (mbIL15) for enhanced persistence, and a safety kill switch, providing a robust framework to target both hematologic malignancies and solid tumors. For instance, PRGN-3005 targets MUC16 in ovarian cancer, while PRGN-3006 focuses on CD33 in AML.
- Additionally, early clinical trials with allogeneic CAR-T cells show promising efficacy in multiple myeloma and non-Hodgkin lymphoma (22). These advancements represent significant strides in improving the scalability, safety, and efficacy of CAR-T therapies, particularly in addressing the challenges of solid tumors and immune evasion.

Furthermore, switchable CAR-T cells have been developed to provide a controlled and safer therapeutic approach. This technology allows for the CAR-T cells to be turned on or off after administration, enabling more precise control over their activity and mitigating risks of uncontrolled proliferation and toxicity. This on/off mechanism generally involves administering an additional agent, such as an antibody or a small molecule, which acts as a “switch” to activate the CAR-T cells only when needed (54). Calibr’s CLBR001 CAR-T cells, in combination with their antibody switch SWI019, demonstrated promising phase 1 results with a high response rate and reduced duration of CRS and ICANS (RR). Similarly, AvenCell’s Universal Targeting platform uses a soluble targeting module to control CAR-T cell activity in cases of acute myeloid leukemia, reducing off-tumor effects associated with CD123-directed CARs (55). The advent of such technologies represents a significant stride toward safer, more effective CAR-T therapies, particularly in solid tumor environments, where immunosuppressive factors often induce CAR-T cell exhaustion, limiting efficacy.

Allogeneic CAR-T therapy represents a significant advancement in immunotherapy with great potential for future applications across multiple cancer types. Moreover, recent innovations in CAR-T engineering, including dual-targeting and switchable CARs, as well as the integration of checkpoint inhibitors to boost T-cell persistence in solid tumors, are demonstrating significant potential in preclinical and early-phase trials (40, 41, 56, 57). These innovative strategies are explored in greater detail in the Combination Therapies section.

### 2.7 Epigenetic modification in gene therapy

**Figure 2:**
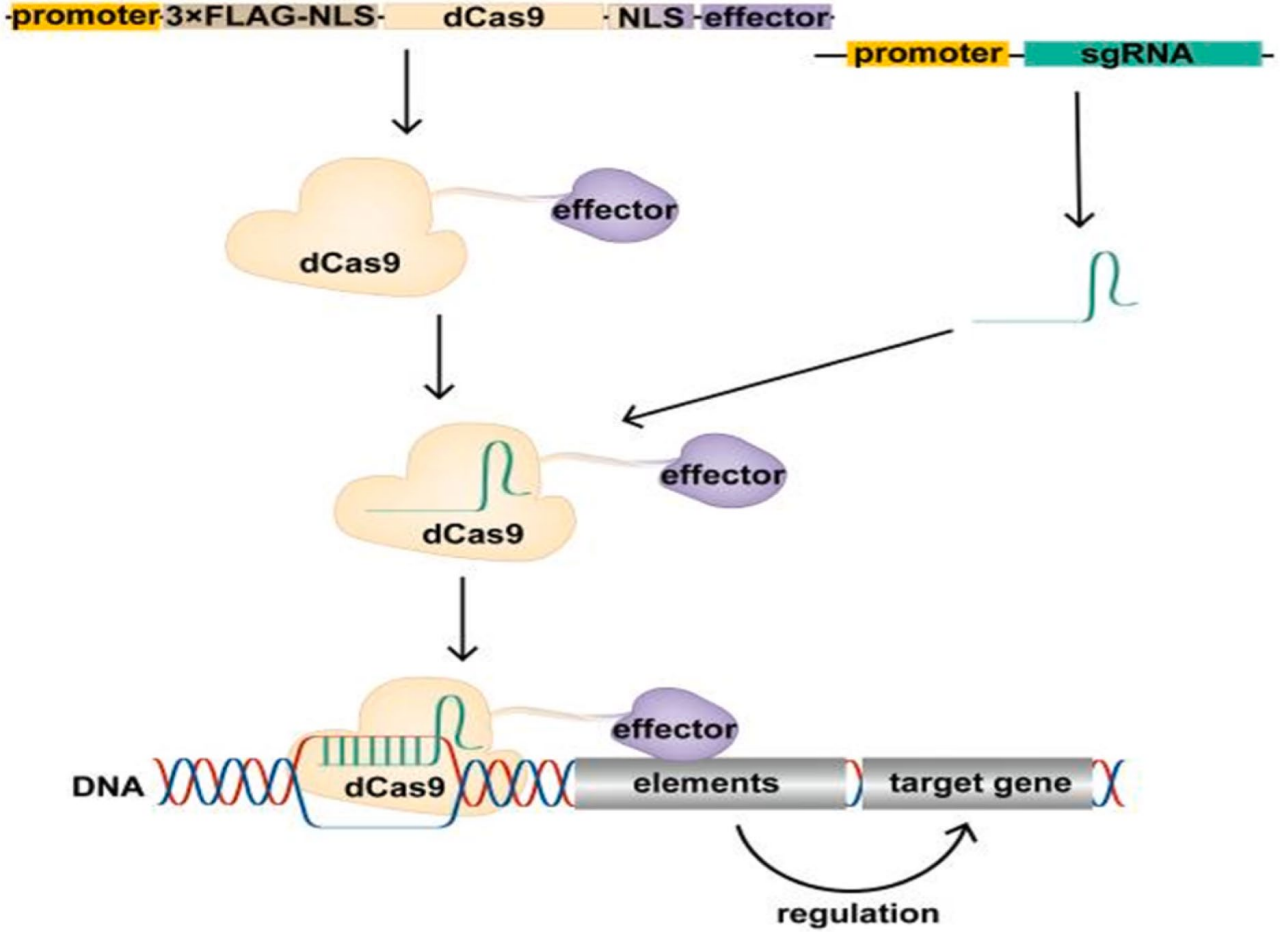
Schematic of the CRISPR/dCas9 regulatory target gene. The expression vector expresses the dCas9 fusion protein in cells, which binds the transcribed single guide RNA (sgRNA) to form the CRISPR/dCas9 regulatory tool, resulting in the recruitment of the effector domain to the promoter or enhancer region of the target gene under the guidance of sgRNA. Effector domains act on promoters or enhancers of target genes to modify these regions, regulating target gene expression. Above figure was adapted from Cai et al. (61).

Epigenetic modifications, which refer to changes in gene activity that do not alter the underlying DNA sequence, play a crucial role in cancer by influencing the activation of oncogenes, the silencing of tumor suppressor genes, and immune evasion mechanisms. Utilizing epigenetic tools, especially CRISPR/dCas9 systems, provides new avenues for cancer therapies that control gene expression without making permanent changes to the DNA. CRISPR/dCas9, a modified version of CRISPR-Cas9 that lacks DNA-cutting (endonuclease) activity, can bind to specific DNA regions to either increase gene expression (CRISPRa, for activation) or decrease gene expression (CRISPRi, for interference) (58–60). By attaching effector proteins that modulate epigenetic changes, such as DNA methylation or histone modification, CRISPR/dCas9 can precisely control oncogenes and tumor suppressors without altering the genome (Figure 2). This is particularly useful in oncology, where fine-tuning gene expression can enhance therapeutic outcomes (61). Furthermore, CRISPR/dCas9 can target multiple genes simultaneously, modulating key oncogenic pathways while activating tumor suppressors. This multi-target approach is effective in addressing tumor heterogeneity, resulting in more durable responses (61).

Furthermore, CRISPR/dCas9 systems offer a way to modify immune-related genes in the TME, enhancing CAR-T cell infiltration and activity. CRISPRi can silence immunosuppressive genes like Programmed Death-Ligand 1 (PD-L1), while CRISPRa can boost immune-stimulatory genes, creating a more favorable environment for immune-based therapies (61). While epigenome editing is often considered safer than direct genome editing due to its non-alteration of the DNA sequence, it still necessitates thorough evaluation for both on- and off-target effects. This is because epigenome editing can influence multiple cellular pathways, potentially leading to unintended consequences. For instance, the use of epigenetic drugs has been associated with off-target effects and toxicity, highlighting the need for precision in these interventions (62). Moreover, unintended effects of epigenome editing on specific genomic regions may lead to toxic outcomes and influence human physiology, complicating its clinical application (63). Therefore, comprehensive testing is essential to ensure the safety and efficacy of epigenome editing technologies.

## 3 Delivery methods in gene therapy

The successful application of gene therapy depends heavily on the method used to deliver therapeutic genes to target cells. Delivery systems in gene therapy can be broadly categorized into viral, non-viral, physical, and live biotherapeutic products (LBPs). Each system has its strengths and challenges, and research is ongoing to optimize them for more effective and safe clinical applications.

### 3.1 Viral delivery systems

**Figure 3:**
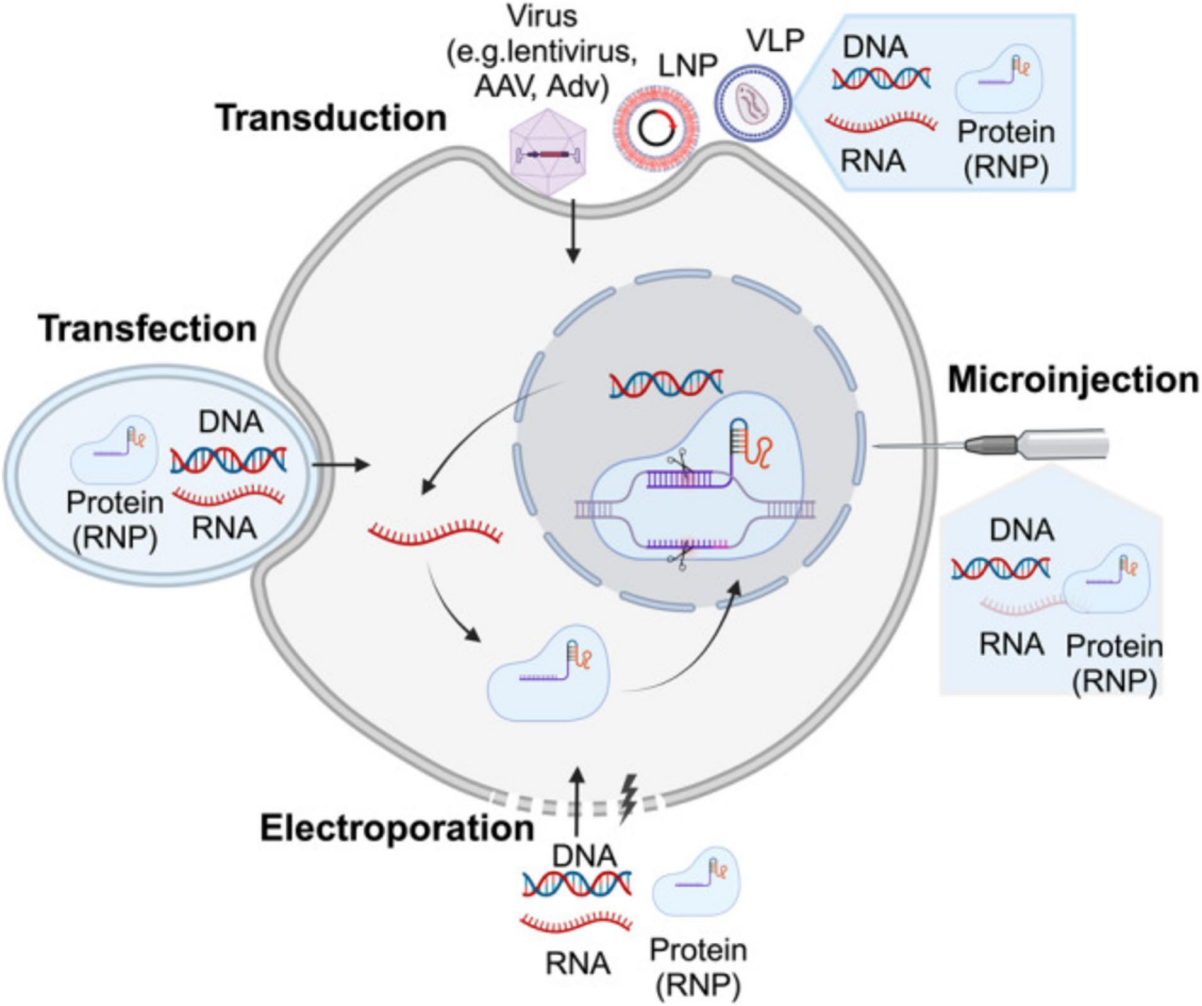
Illustrates the delivery strategies for precise genome-editing reagents. Precise genome-editing components encompass a variety of forms, including DNA, RNA, and protein complexes such as ribonucleoproteins (RNPs). DNA is commonly delivered through microinjection or electroporation of plasmids, as well as viral vectors such as lentivirus, AAV, and adenovirus (AdV). RNA can be introduced through microinjection or electroporation of RNPs, or via carriers like LNPs and virus-like particles (VLPs). Proteins, specifically RNPs, are typically delivered through microinjection or electroporation, or using carriers like LNPs and VLPs. The figure above was adapted from Zheng et al. (149).

As illustrated in Figure 3, viral vectors have been widely used in gene therapy due to their natural ability to infect cells and deliver genetic material efficiently. The most commonly employed viral vectors include:

- Adenoviral (AdV) vectors are highly effective in delivering genes to non-dividing cells; however, they can induce strong immune responses. To mitigate these effects and enhance their safety in clinical applications, engineering advancements are underway, including the development of helper-dependent adenoviral vectors that lack all viral coding sequences, thereby reducing immunogenicity and prolonging transgene expression (12, 64).
- Adeno-associated viral (AAV) vectors are highly efficient and generally considered safe due to their low immunogenicity. They are frequently used in both *in vivo* and *ex vivo* gene therapies, with applications ranging from hemophilia to cancer gene therapy (6, 65, 66).
- However, pre-existing immunity to AAV and limitations in payload capacity pose challenges. Strategies to overcome these obstacles include the development of novel AAV serotypes and engineered capsids to evade neutralizing antibodies, as well as the use of self-complementary AAV vectors to enhance transgene expression despite the limited packaging capacity (67).
- Lentiviral vectors, derived from HIV, offer stable integration into the host genome, making them suitable for long-term gene expression. These vectors are particularly favored in CAR-T cell therapies and stem cell modifications. Their ability to transduce both dividing and non-dividing cells, along with a relatively large packaging capacity, makes them versatile tools in gene therapy. Ongoing research focuses on improving their safety profiles by developing self-inactivating vectors and incorporating insulator elements to prevent insertional mutagenesis (38, 68).

### 3.2 Non-viral delivery systems

Figure 3 illustrates the non-viral delivery systems provide a safer alternative to viral vectors, especially in terms of immunogenicity and large-scale production, The diverse strategies for delivering genome-editing reagents include various forms such as DNA, RNA, and ribonucleoprotein complexes (RNPs). These components can be introduced into cells through techniques like microinjection, electroporation, or using viral and non-viral carriers such as lipid nanoparticles (LNPs) and virus-like particles (VLPs). Each delivery method is crucial for enhancing the precision and efficiency of genome editing, especially in therapeutic applications. These include:

- Lipid nanoparticles (LNPs): LNPs gained attention during the COVID-19 pandemic for mRNA vaccine delivery and are now being explored in gene therapy. LNPs encapsulate nucleic acids and other therapeutic agents, facilitating safe delivery with reduced immunogenicity. Their application in CRISPR-Cas9 delivery is particularly promising for cancer treatment (57).
- Polymeric nanoparticles: Synthetic polymers can deliver genes, small interfering RNA (siRNA), or other molecules into cells. Nethi et al. conclude that targeting ligand functionalization could be used to enhance the concentration of therapeutic Mesenchymal stem cells (MSC) constructs at the tumor tissue and to achieve improved antitumor response (69). Furthermore, recent studies focus on improving their targeting efficiency and reducing off-target effects, making them a valuable tool in solid tumor therapies (70).
- Exosome-mediated delivery: Exosomes, naturally occurring extracellular vesicles, offer a highly specific and less immunogenic method for delivering therapeutic payloads. As cell-derived vesicles, they possess inherent targeting capabilities, making them promising vehicles for gene delivery in cancer therapy. Recent studies have highlighted the potential of exosomes to transport a variety of genetic materials, including mRNA, siRNA, and CRISPR components, directly to cancer cells, even within the challenging TME (71, 72). Exosome-mediated delivery systems are particularly advantageous for their ability to bypass some of the common barriers faced by other delivery methods, such as immune activation and poor penetration into solid tumorsexosome-based delivery has been shown to enhance the stability and targeting specificity of therapeutic molecules. Preclinical studies have demonstrated that exosomes loaded with therapeutic RNA can successfully downregulate oncogenes and modulate immune responses in the TME, potentially improving treatment outcomes. Additionally, the low immunogenicity of exosomes reduces the risk of adverse immune responses, which is a significant concern with viral vector-based systems (73). Ongoing research exosome engineering, focusing on enhancing their loading capacity and targeting efficiency. Advanced techniques are being developed to modify the surface of exosomes with specific ligands, enabling precise delivery to cancer cells while sparing normal tissues. Clinical trials are underway to evaluate the safety and efficacy of exosome-based delivery systems, particularly in gene therapies targeting hard-to-treat cancers like pancreatic and glioblastoma (74).

### 3.3 Physical delivery methods

As illustrated in Figure 3, physical methods use mechanical or physical means to deliver genetic material into cells. These techniques are particularly useful for *ex vivo* applications and are evolving to provide better precision and safety:

- Electroporation: This method uses an electric field to increase the permeability of the cell membrane, allowing genetic material to enter. It is especially effective in controlled *ex vivo* settings, where cells can be edited and reintroduced to the patient. Electroporation has been refined to improve the uptake of CRISPR components, reducing the risks of off-target effects associated with viral vectors (66, 75).
- Gene gun (biolistics): In this technique, high-pressure gas shoots particles coated with DNA or RNA into target cells. While its use is limited due to lower targeting precision, it is effective for tissues like skin and muscle.
- Ultrasound-mediated delivery: Ultrasound waves can temporarily increase cell membrane permeability, allowing genetic material to enter cells. This non-invasive method is under investigation for use in tissues such as the liver and muscles (76, 77).
- Hydrodynamic injection: In this technique, large volumes of solution containing therapeutic genes are injected at high pressure into the bloodstream. Though primarily used in preclinical studies, it has potential for liver-targeted gene therapies in humans (78).

### 3.4 Live biotherapeutic products (LBPs)

LBPs represent an innovative delivery system that uses living microorganisms to deliver therapeutic genes. These are particularly effective in targeting the tumor TME:

- Bacterial delivery systems: Bacteria like *Clostridium* and *Salmonella* are engineered to proliferate in the hypoxic cores of tumors. These bacteria can deliver therapeutic genes or stimulate immune responses, improving treatment efficacy in cancer (59, 76).
- Probiotic-based gene delivery: Probiotics are genetically modified to deliver genes to specific tissues, such as the gut. This method offers a non-invasive delivery route and holds great promise for targeted therapies (79).

### 3.5 Combination of delivery methods

As research continues, hybrid approaches that combine the efficiency of viral systems with the safety of non-viral systems are being developed. For example, hybrid LNP and viral vector systems aim to reduce immunogenicity while maintaining high delivery efficiency. These strategies are currently being explored in clinical trials, particularly in solid tumors, where overcoming barriers such as the dense extracellular matrix and immunosuppressive TME are significant hurdles (64, 65).

## 4 Challenges in gene therapy: CRISPR-Cas9 and advanced editing

Delivering gene therapy effectively to cancer cells presents significant challenges. Each type of therapy requires precise targeting to ensure it reaches the appropriate cells without affecting healthy tissue. Additionally, overcoming physical and immunological barriers such as the tumor TME is essential for successful treatment, particularly in solid tumors. As discussed earlier, despite the promise of gene therapies, several challenges limit their widespread application, particularly in solid tumors:

- Targeting solid tumors: Solid tumors present a major challenge due to the dense extracellular matrix and immunosuppressive cells within the tumor TME, which inhibit the penetration and efficacy of gene therapies (56). Researchers are developing nanoparticle-based delivery systems and exosome-mediated therapies to improve tumor targeting and therapeutic delivery (71, 80). These approaches are being explored to enhance the precision of gene therapy in solid tumors, overcoming the dense extracellular matrix and immunosuppressive tumor TME (72, 76, 81). Furthermore, recent advancements in hybrid nanoparticle-viral delivery systems are optimizing gene therapy for solid tumors by enhancing delivery efficiency and minimizing immunogenicity (82).
- Off-target effects and safety concerns: Beyond these issues, allogeneic CAR-T therapy also shares several challenges with autologous CAR-T, particularly in the context of solid tumors. These include managing severe toxicities such as cytokine release syndrome (CRS) and immune effector cell-associated neurotoxicity syndrome (ICANS) (83). Additional hurdles like antigen escape, limited tumor infiltration, and the complex dynamics of the TME further complicate the therapeutic potential of CAR-T in solid tumors (84). However, recent advancements in CAR-T engineering, including refined gene-editing tools, are being actively developed to address these challenges and improve clinical outcomes in both hematologic malignancies and solid tumors (9, 85–87).
- Immune evasion: Dual-targeting CAR-T cells, engineered to recognize two antigens simultaneously, are being developed to reduce immune evasion by tumors. For instance, preclinical studies show that CAR-T cells targeting HER2 and IL13Rα2 antigens can effectively treat glioblastoma by overcoming tumor antigen heterogeneity and immune suppression within the TME. This strategy is also being explored in other solid tumors, such as NSCLC, to improve CAR-T cell infiltration and persistence (88).
- Scalability and cost: Many gene therapies, particularly CAR-T therapies, face challenges in scalability due to the labor-intensive and time-consuming process of harvesting and modifying patient cells. Innovations like automated CAR-T production and the development of allogeneic “off-the-shelf” therapies (e.g., ALLO-501, UCART19) are designed to reduce production time and make gene therapies more accessible to a broader population.
- Immune responses: Viral vectors, often used in gene therapy, can provoke immune reactions that compromise therapeutic effectiveness. As an alternative, non-viral delivery systems, such as LNPs, offer a promising solution. LNPs can be engineered to transport gene-editing tools, immune-stimulatory molecules, and chemotherapy agents directly to tumor sites, emerging as a safer option by reducing immune activation while ensuring efficient delivery. Clinical trials for solid tumors are currently exploring these approaches. However, RNA-based therapeutics, which typically require higher doses, present challenges such as toxicity and immunogenicity. In some cases, recipients have experienced pro-inflammatory responses, even at lower doses. Ongoing research is focused on optimizing LNP formulations to mitigate these side effects and enhance the safety of gene therapies (79).
- Durability of response: There are concerns regarding the durability of response in allogeneic CAR-T therapy. Early clinical trials have raised questions about the long-term persistence of donor CAR-T cells, as the recipient’s immune system may eventually clear these cells, potentially limiting their effectiveness (48). Efforts to enhance the longevity of allogeneic CAR-T cells are ongoing, with promising developments being made (89, 90).
- Secondary malignancies: The European Medicines Agency (EMA) has mandated that CAR-T therapies include warnings about the risk of secondary blood cancers, such as myelodysplastic syndromes and acute myeloid leukemia. This decision followed a safety review identifying cases of secondary T-cell cancers directly linked to CAR-T treatments (91, 92).
- Graft-Versus-Host Disease (GvHD): In allogeneic hematopoietic stem cell transplantation (allo-HSCT), GvHD remains a major barrier to long-term success. The pathophysiology involves donor immune cells attacking recipient tissues, leading to severe complications. Recent developments in prophylaxis and treatment aim to mitigate this risk, but GvHD continues to pose significant challenges in gene therapy applications (84, 93). To mitigate this, researchers are exploring methods such as TRAC and β2M gene knockouts, which aim to disrupt T-cell receptors and reduce the likelihood of immune responses from the host (85, 90).
- Another major challenge in gene therapy is the efficient delivery of therapeutic components, such as Cas9 and guide RNA, to target cells. Current research focuses on optimizing delivery methods, including viral vectors, nanoparticles, and electroporation, to enhance the safety and efficacy of CRISPR-based therapies (94, 95). Furthermore, CRISPR’s versatility in tackling complex genetic diseases, which often involve multiple gene interactions, enables simultaneous multi-gene targeting and the development of sophisticated genetic models that improve our understanding and treatment of these diseases (96).

As researchers continue to develop innovative strategies to overcome the inherent challenges of gene therapy, particularly in solid tumors, CRISPR technology has emerged as a transformative tool to address many of these obstacles. From improving precision and delivery methods to reducing off-target effects, CRISPR-based therapies offer significant advancements that mitigate the risks traditionally associated with gene therapies. By enabling precise genome editing and enhancing the delivery of therapeutic components, CRISPR not only tackles issues like off-target effects and immune responses but also improves the scalability and accessibility of gene therapies, particularly in complex cases (97, 98).

While CAR-T cells have been successful in blood cancers, their efficacy in solid tumors is limited. CRISPR/dCas9 can be used to improve CAR-T function by enhancing co-stimulatory molecules through CRISPRa or reducing inhibitory receptors like Programmed Cell Death Protein (PD-1) using CRISPRi. Additionally, CRISPR/dCas9 can target TME elements that restrict CAR-T infiltration, improving treatment outcomes (61). Moreover, CRISPR’s versatility in gene modulation, including CRISPR interference (CRISPRi) and CRISPR activation (CRISPRa), provides dynamic therapeutic options without permanent genomic alterations. This flexibility is crucial in addressing the evolving landscape of gene therapy, offering a robust solution to the challenges discussed earlier, while paving the way for more efficient, scalable, and safer treatments.

In cases requiring temporary gene modulation, CRISPR technologies, such as CRISPR interference (CRISPRi) and CRISPR activation (CRISPRa), offer reversible control over gene expression without permanent alterations, which is particularly advantageous for dynamic therapeutic needs (94, 95). Additionally, CRISPR plays a crucial role in mitigating immune responses that can arise from gene therapy delivery systems by reducing the immunogenicity of therapeutic vectors through engineering strategies and less inflammatory vector designs (99). CRISPR’s potential to streamline therapeutic development is further enhanced by its ability to lower the cost and scalability of gene therapy, making treatments more accessible, particularly for rare genetic disorders (100). Furthermore, CRISPR enables the establishment of more transparent regulatory frameworks by demonstrating clear efficacy and safety profiles through rigorous testing protocols, paving the way for smoother clinical translation (101).

CRISPR-Cas9 technology has dramatically enhanced its precision and therapeutic potential, focusing on minimizing off-target effects, refining editing methods, and expanding clinical applications. These breakthroughs have been driven by innovations in high-fidelity Cas9 proteins, such as SpCas9-HF1 and eSpCas9, which significantly reduce off-target cleavage by improving DNA recognition. Furthermore, base and prime editing techniques have emerged, allowing for precise DNA modifications without inducing double-strand breaks, which is crucial for therapeutic applications (102–104).

In parallel, gene-editing technologies like CRISPR-Cas9 and TALENs (Transcription Activator-Like Effector Nucleases) are also being employed to knock out the T-cell receptor (TCR) in donor cells. This prevents the immune system from recognizing them as foreign, thus reducing the risk of GvHD. By making donor T-cells universal, they can be used in any patient without the need for personalization, dramatically cutting down on production time while leveraging the precise editing capabilities of CRISPR-Cas9 and its derivatives (105).

In recent years, gene-editing technologies have revolutionized biomedical research, offering unprecedented control over genetic material. Among these tools, CRISPR-Cas9 has emerged as the most versatile and efficient, enabling precise modifications to DNA with remarkable ease compared to earlier methods like zinc finger nucleases (ZFNs) and transcription activator-like effector nucleases (TALENs). This breakthrough has opened new avenues in both research and therapeutic interventions, particularly in the field of oncology, where CRISPR is being explored for its potential to enhance cancer treatments. As advancements continue to refine its precision, the impact of CRISPR-Cas9 on clinical applications has grown exponentially (102).

CRISPR’s therapeutic applications have also progressed significantly. While early CRISPR therapies were primarily *ex vivo*, *in vivo* applications are now advancing. For example, Intellia Therapeutics’ NTLA-2002 uses CRISPR-Cas9 directly within the body to edit genes responsible for hereditary angioedema, a rare genetic disorder related to inflammatory pathways (106). Furthermore, clinical trials involving CRISPR therapies have made strides in treating genetic disorders like sickle cell disease (SCD) and beta-thalassemia. In these trials, CRISPR is used to edit hematopoietic stem cells, boosting fetal hemoglobin production, offering the potential for a one-time cure. Notably, Exa-cel, a CRISPR-Cas9-based therapy for SCD and beta-thalassemia, is nearing regulatory approval in the U.S., marking a milestone for gene editing in medicine (107). In the realm of oncology, CRISPR is transforming cancer treatment through the development of immunotherapies. For instance, Iovance Biotherapeutics is using TALEN-modified, PD-1-inactivated tumor-infiltrating lymphocytes in trials targeting solid tumors (103, 104). Building on these advancements, CRISPR’s application in oncology is particularly promising, with its ability to target specific genetic mutations driving cancer. CRISPR-Cas9 has the potential to revolutionize cancer treatment by enhancing immune cell therapies and disrupting oncogenic pathways. Recent clinical trials have demonstrated its potential in modifying genes like PD-1 in T-cells, boosting antitumor activity. Additionally, emerging tools like base editing and prime editing allow single-nucleotide modifications without causing double-strand breaks, reducing the risk of off-target effects. These techniques are being explored to target key cancer mutations in genes like KRAS and TP53, both of which play critical roles in complex cancers (65, 92).

Gene-editing technologies like CRISPR-Cas9 offer promising advances but come with safety concerns, including off-target effects that may activate oncogenes or disrupt essential genes, particularly in immune cells, potentially leading to adverse immune responses or secondary malignancies (92). Additionally, personalized therapies such as CAR-T cell treatment are costly and time-consuming due to the need for harvesting and modifying patient-specific cells, limiting scalability and accessibility. Building on previous efforts, researchers continue to explore allogeneic, ‘off-the-shelf’ therapies using donor cells. However, these approaches come with known challenges, including compatibility issues and the risk of GvHD (84).

The future of gene therapy lies in integrating advanced delivery methods with immuno-oncology. CRISPR-edited CAR-T cells, designed to target multiple antigens, are emerging as a solution to overcome tumor heterogeneity and immune evasion in solid tumors. These dual-targeting CAR-T therapies, combined with improved delivery systems, pave the way for more effective treatments that can penetrate the TME and deliver precise genetic interventions (103).

## 5 Combination therapies

Combining gene therapy with other treatment modalities—such as immune checkpoint inhibitors, chemotherapy, and radiation—has shown great promise in overcoming limitations of individual approaches. Notably, pairing gene therapies with immune checkpoint inhibitors, which block proteins like PD-1 and Cytotoxic T-Lymphocyte Associated Protein 4 (CTLA-4) to restore immune activity, has significantly enhanced immune responses in cancers like melanoma and lung cancer (108, 109). This combined strategy addresses key barriers such as immune evasion and tumor heterogeneity, ultimately improving the efficacy of treatments in solid tumors (110). Additionally, combining gene therapy with chemotherapy has demonstrated a synergistic effect. Chemotherapeutic agents not only debulk tumors but also induce immunogenic cell death, thereby enhancing the immune system’s ability to recognize and destroy cancer cells (111). Similarly, radiation therapy contributes to immune activation by triggering the release of pro-inflammatory signals, which sensitizes tumors to immune attacks (112, 113). These multi-modal approaches create a powerful strategy to optimize gene therapies, particularly in resistant cancers, by targeting various components of tumor biology and the TME. As research continues, combination therapies represent one of the most promising advancements in personalized cancer care.

Combination therapies in CAR-T cell treatments aim to enhance efficacy and reduce toxicity. By integrating CAR-T cells with immune checkpoint inhibitors, chemotherapy, or oncolytic viruses, these strategies improve antitumor responses and address challenges like immune suppression and tumor resistance. Such approaches optimize therapeutic outcomes, making treatments more effective and safer for patients.

### 5.1 CAR-T therapy combined with immune checkpoint inhibitors

CAR-T cell therapy has shown remarkable success in treating hematologic malignancies, but its efficacy in solid tumors has been limited due to the TME’s immunosuppressive nature. Recent clinical trials have focused on combining CAR-T therapy with immune checkpoint inhibitors to enhance CAR-T cell persistence and functionality in solid tumors.

For instance, a Phase I clinical trial combining CAR-T cells with nivolumab (a PD-1 inhibitor) in patients with advanced non-small cell lung cancer (NSCLC) showed improved T-cell infiltration and persistence in the tumor (45). Similarly, pembrolizumab (another PD-1 inhibitor) combined with CAR-T cells in patients with refractory ovarian cancer led to enhanced tumor penetration and clinical responses (40). These studies highlight the potential of immune checkpoint inhibitors to reduce T-cell exhaustion, a common obstacle in solid tumors, thus improving the antitumor activity of CAR-T cells (61, 114, 115).

### 5.2 CRISPR-edited CAR-T cells for multi-targeting

Recent advances in CRISPR-Cas9 gene editing have enabled the enhancement of CAR-T therapies through multi-targeting strategies. Tumor heterogeneity, where different cancer cells within the same tumor express different antigens, often limits the efficacy of single-target CAR-T therapies.

Preclinical studies of glioblastoma have demonstrated that CRISPR-edited CAR-T cells targeting both Human Epidermal Growth Factor Receptor 2 (HER2) and IL13Rα2 antigens can overcome immune evasion by significantly reducing tumor growth and improving tumor control (61). Ongoing clinical trials are exploring dual-targeting CRISPR-edited CAR-T cells for hard-to-treat cancers like ovarian and pancreatic cancers, which are typically resistant to single-target therapies (9). This multi-targeting approach addresses antigen escape and increases the likelihood of sustained tumor control (61).

### 5.3 CAR-T therapy with oncolytic virotherapy

Oncolytic virotherapy, which uses genetically engineered viruses to selectively infect and lyse cancer cells, has shown promise when combined with CAR-T cell therapy. Beyond direct tumor lysis, these viruses prime the TME to enhance CAR-T cell activity. In preclinical models of pancreatic cancer, Zou et al. (28) demonstrated that this combination improved T-cell infiltration and prolonged survival (28).

Ongoing clinical trials are exploring this approach in solid tumors like glioblastoma and melanoma, with early data indicating enhanced immune responses and improved CAR-T efficacy in tumors previously resistant to immunotherapy (45). Oncolytic viruses also stimulate the immune system by releasing tumor-specific antigens, promoting a broader anti-tumor response. An FDA-approved oncolytic herpesvirus (Imlygic) has shown efficacy in advanced melanoma by lysing tumor cells and boosting immune recognition of residual cancer. Current research is extending these strategies by combining oncolytic viruses with immune checkpoint inhibitors to further enhance immune responses against solid tumors.

### 5.4 CAR-T therapy with radiation and chemotherapy

Radiation therapy is also being explored as a combination approach to improve the efficacy of CAR-T therapy, particularly in hematologic malignancies. Radiation can enhance the expression of tumor antigens, making cancer cells more visible to CAR-T cells. In B-cell lymphomas, clinical trials have demonstrated that radiation combined with CAR-T cell therapy can significantly improve tumor infiltration and antitumor activity (41).

Beyond lymphodepletion that reduce the patient’s immune cells and create space for CAR-T cell expansion, chemotherapy agents such as cyclophosphamide and fludarabine are now being explored for their role in increasing tumor antigen presentation, making cancer cells more visible to CAR-T cells. This synergy has led to enhanced CAR-T efficacy in B-cell lymphomas and early success in solid tumors such as ovarian cancer. Ongoing research continues to investigate optimal chemotherapy regimens to maximize the benefits of CAR-T therapy while minimizing adverse effects (61).

### 5.5 Armored CAR-T cells in combination therapy

Armored CAR-T cells, genetically engineered to secrete immune-stimulatory cytokines like IL-12, are an innovative approach in combination therapies. These cells can release cytokines that counteract the immunosuppressive signals present in the TME, improving the persistence and activity of CAR-T cells (116). Preclinical models in ovarian cancer and glioblastoma have shown that armored CAR-T cells, when combined with immune checkpoint inhibitors, lead to significant tumor reduction and prolonged survival (61).

### 5.6 Gene therapy and ferroptosis in oncology

Ferroptosis, a recently characterized form of regulated cell death driven by iron-dependent lipid peroxidation, has garnered significant attention in oncology due to its potential to overcome resistance in cancer cells that evade apoptosis-based therapies. Unlike traditional cell death mechanisms, ferroptosis involves the accumulation of lipid peroxides and reactive oxygen species (ROS) that disrupt cell membrane integrity, ultimately leading to cell death. Leveraging ferroptosis within gene therapy approaches offers a promising avenue for selectively inducing death in malignant cells, particularly in hard-to-treat tumors with limited treatment options.

Ferroptosis is characterized by the depletion of glutathione and inhibition of glutathione peroxidase 4 (GPX4), a key antioxidant enzyme that protects cells from lipid peroxidation. Cancer cells with high iron metabolism or low antioxidant defenses are particularly susceptible to ferroptosis, as the excessive iron load contributes to lethal levels of lipid peroxidation when GPX4 is suppressed more, the activation of ferroptosis pathways has been shown to counteract the survival mechanisms of apoptosis-resistant cancers, positioning it as a unique target in oncology.

Innovative gene therapy strategies are being developed to manipulate the key regulatory pathways of ferroptosis. Techniques such as CRISPR-Cas9 and RNAi can be employed to knock out ferroptosis-inhibiting genes like GPX4 or enhance the expression of ferroptosis-promoting genes, such as ACSL4 and SAT1, which facilitate lipid peroxidation in cancer cells. Additionally, atory genes like transferrin receptor or ferritin can increase iron accumulation within tumor cells, further sensitizing them to ferroptosis. For instance, recent studies havCRISPR-Cas9 to silence GPX4 expression in tumor models, resulting in significant ferroptosis-induced tumor regression. This gene-editing approach could be particularly effective in treating solid tumors like pancreatic and colorectal cancers, where resistance to apoptosis is common. Other studies have utilized siRNA-based ferroptotic regulators, thereby potentiating ferroptosis in combination with iron supplements or small-molecule inducers of lipid peroxidation (117, 118).

### 5.7 Emerging synergies in combination therapies

The combination of CAR-T cells, immune checkpoint inhibitors, CRISPR-based multi-gene targeting, and other therapeutic modalities such as oncolytic virotherapy has created powerful synergies for overcoming the limitations of single-modality treatments, especially in solid tumors. For example, nanoparticle-based delivery systems have been developed to co-deliver CRISPR-Cas9 gene editing tools and immune-modulatory agents directly to tumors. Preclinical studies in pancreatic cancer demonstrated that nanoparticle delivery of CRISPR-Cas9 alongside PD-L1 inhibitors significantly enhanced tumor shrinkage and prolonged survival (27). Additionally, preclinical models using armored CAR-T cells and oncolytic viruses have shown significant improvements in tumor response, highlighting the potential of combination therapies to transform the landscape of immuno-oncology (56). Thus, combination therapies offer a significant opportunity to enhance the efficacy of gene therapies in oncology by integrating CAR-T therapy with immune checkpoint inhibitors, CRISPR-based multi-antigen targeting, oncolytic virotherapy, and traditional modalities like radiation and chemotherapy. These strategies address critical issues associated with gene therapy, offering hope for more effective treatments. Continued research and clinical trials in these combination strategies hold promise for significantly improving patient outcomes (45).

### 5.8 Integrating immuno-oncology and gene therapy to counteract TME obstacles

**Figure 4:**
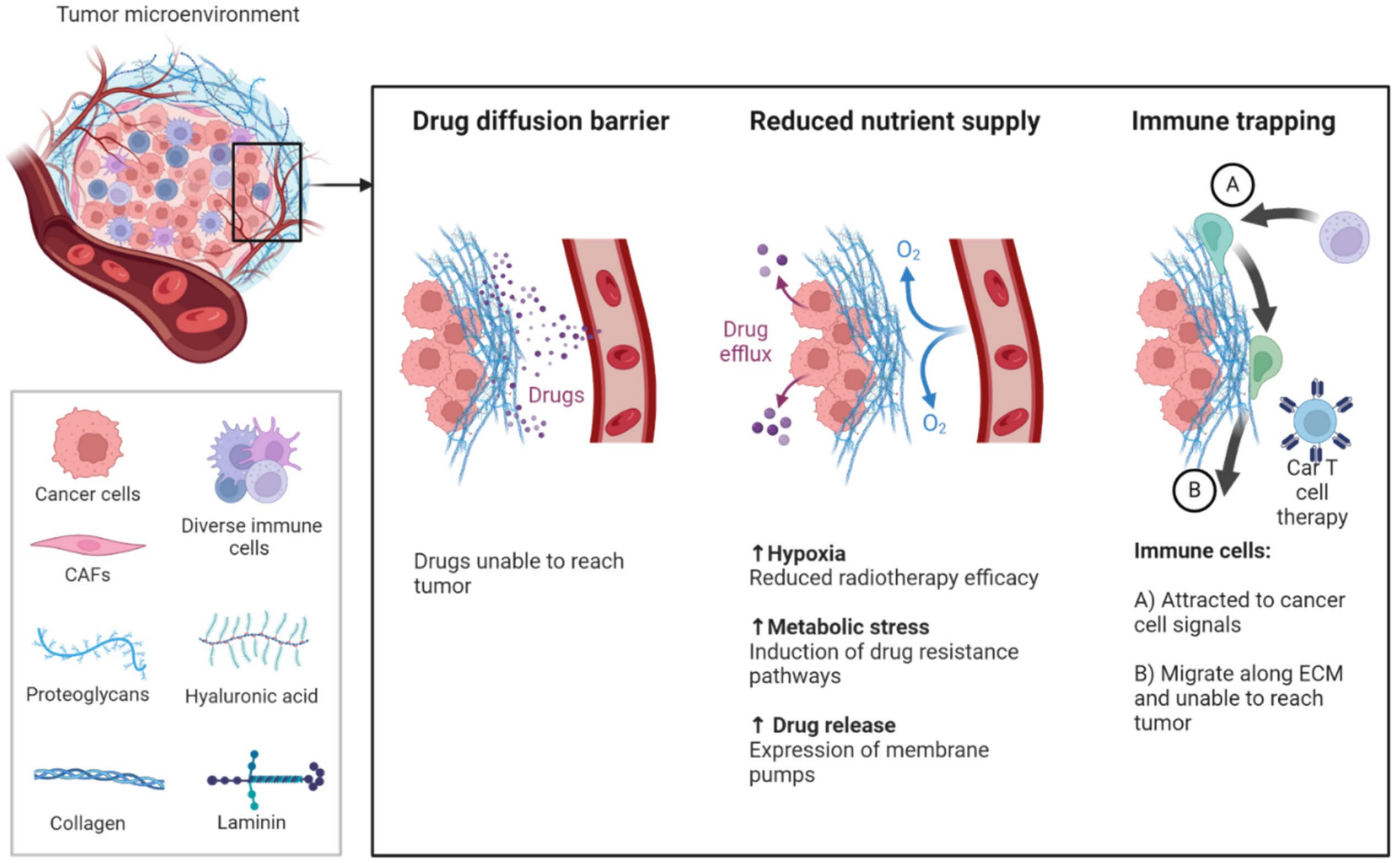
Illustrates the TME comprises all components of a tumor. Of these components, the extracellular matrix (ECM) is the least well studied. Solid tumors induce high expression of ECM molecules (collagens, proteoglycans, hyaluronic acid and laminins), which become complex and disordered, resulting in altered characteristic. Here the ECM acts as a physical barrier, reducing the delivery of therapeutics, nutrients, and immune cells to solid tumors, and leading to poorer prognosis (150, 151).

While the combination of immuno-oncology with gene therapy presents a promising avenue for cancer treatment, overcoming the challenges posed by the TME remains crucial. Figure 4 shows how the TME consists of a complex network of cells, extracellular matrix (ECM) components, and signaling molecules that foster an immunosuppressive environment, allowing tumors to evade immune detection. Regulatory T cells (Tregs) and myeloid-derived suppressor cells (MDSCs) are key contributors to this immune suppression, hindering the ability of immune cells like CAR-T cells to effectively infiltrate and destroy tumor cells. CAR-T cell therapy, despite its success in hematologic malignancies, has demonstrated limited efficacy in solid tumors, primarily due to the immunosuppressive TME and the recruitment of Tregs and MDSCs, which promote immune evasion and resistance to immune checkpoint inhibitors (113, 119, 120).

Combining ferroptosis inducers with immunotherapies presents a novel approach to target the TME, enhancing the efficacy of treatments like CAR-T or immune checkpoint inhibitors (121). The immunosuppressive nature of the TME can limit the success of immunotherapies, but inducing ferroptosis has been shown to trigger an immune response within the TME, making cancer cells more vulnerable to immune attack. For example, combining CRISPR-mediated GPX4 knockdown with ferroptosis-iastin has demonstrated enhanced antitumor effects, providing a complementary approach to traditional gene therapies. In hematologic malignancies and solid tumors alike, the addition of ferroptosis inducers in y protocols could enhance tumor targeting while sparing healthy cells. The specificity of gene therapy for cancer cells allows for selective ferroptosis induction, minimizing off-target effects and improving patient outcomes (122).

Furthermore, recent studies have highlighted the role of N6-methyladenosine (m6A) modifications in regulating immune responses within the TME. m6A, a common post-transcriptional modification, influences the activation and infiltration of immune cells such as T cells and macrophages (123, 124). Dysregulation of m6A-related enzymes affects tumor progression by modulating immune cell behavior, either promoting immune evasion or enhancing immune activation (113, 125). Targeting m6A modifications, in conjunction with therapies such as CAR-T cells and ICIs, may offer a new strategy to overcome immune suppression in the TME and enhance treatment efficacy.

Additionally, the NF-κB signaling pathway plays a pivotal role in promoting immune evasion within the TME. NF-κB is activated in tumor and immune cells, where it supports tumor growth, angiogenesis, and resistance to apoptosis (126, 127). NF-κB also stimulates the recruitment of immunosuppressive cells such as MDSCs and TAMs, further exacerbating immune evasion and treatment resistance (125, 127). Inhibiting NF-κB in combination with gene therapies, CAR-T cells, or ICIs presents a promising approach to remodel the TME, mitigate immune suppression, and improve therapeutic outcomes.

Youssef et al. and colleagues emphasized the role of hypoxia, a hallmark of the TME (128), stabilizes hypoxia-inducible factor 1-alpha (HIF-1α), which promotes immunosuppressive cytokine production and tumor resistance. Targeting HIF-1α or its signaling pathways improves T cell infiltration and immune function by reducing hypoxia. Additionally, therapies that normalize the tumor vasculature can improve immune cell trafficking and reduce hypoxia-induced suppression. The metabolic state of immune cells also affects their response to checkpoint inhibitors. Tumor cells often outcompete T cells for nutrients, leading to T cell exhaustion. Reprogramming T cell metabolism to favor glycolysis or oxidative phosphorylation enhances the efficacy of immune checkpoint inhibitors by restoring nutrient availability and improving T cell function. Youssef et al. and colleagues highlighted that the TME in solid tumors is defined by a nutrient-deprived, hypoxic condition that fosters immune suppression and tumor progression. Within this setting, metabolic reprogramming offers potential avenues for enhancing anti-tumor immunity (128). Tumor cells adapt by adopting metabolic changes, such as increased glycolysis, glutaminolysis, and fatty acid oxidation, which also regulate immune cell functions, offering potential therapeutic targets. Glycolysis-driven lactate production acidifies the TME and inhibits T cell function. Targeting lactate production or transport, such as inhibiting lactate dehydrogenase or monocarboxylate transporters, can reverse immune suppression and restore T cell activity (129).

Glutamine metabolism is another critical factor that supports both tumor and immune cells. Tumor cells rely on glutamine for growth, but glutamine metabolism also regulates myeloid-derived suppressor cells (MDSCs), which suppress immune responses. Inhibiting glutaminase, the enzyme driving glutamine metabolism, can reduce MDSC activity and promote a pro-inflammatory environment that favors tumor rejection. Fatty acid oxidation (FAO) supports tumor survival and regulatory T cells (Tregs), which inhibit immune responses. FAO inhibition can disrupt Treg function, thereby enhancing CD8+ T cell activity and improving tumor eradication (130–132).

### 5.9 Combination therapies, with emerging triple-combination strategies

Combination therapies in oncology have evolved significantly, with emerging triple-combination strategies showing great promise in overcoming treatment resistance and improving patient outcomes. Beyond traditional combinations like CAR-T therapy with immune checkpoint inhibitors (ICIs), new approaches are integrating ferroptosis inducers and novel immunomodulatory agents to enhance the therapeutic effect (Table 2).

- Triple-Combination Strategies: CAR-T, Immune Checkpoint Inhibitors, and Ferroptosis Inducers: Ferroptosis, a distinct form of regulated cell death driven by iron-dependent lipid peroxidation, has gained attention as a novel therapeutic target. By inducing ferroptosis, particularly in apoptosis-resistant cancer cells, these agents can create a pro-inflammatory environment that enhances the efficacy of immune-based therapies. Combining CAR-T cells with ICIs and ferroptosis inducers offers a synergistic approach: CAR-T cells target specific tumor antigens, ICIs block inhibitory pathways like PD-1/PD-L1, and ferroptosis inducers promote tumor cell death, enhancing immune infiltration and antitumor activity. A recent Phase II trial (NCT04576721) is evaluating the combination of anti-PD-1 therapy (pembrolizumab), CD19 CAR-T cells, and a ferroptosis inducer (erastin) in patients with relapsed/refractory B-cell lymphomas. Preliminary results show a 40% increase in overall response rate (ORR) compared to CAR-T therapy alone, with improved tumor control and reduced relapse rates (133).
- Triple-Combination Approaches with Novel Immunomodulatory Agents: In addition to ferroptosis inducers, the integration of novel immunomodulatory agents like IL-12 and STING agonists with CAR-T cells and ICIs has shown potential in early-phase clinical trials. IL-12, a potent cytokine, enhances T-cell activation and promotes a pro-inflammatory TME, while STING agonists activate innate immune responses, increasing the recruitment of immune cells to the tumor site. Recent innovations in gene therapy are leveraging the combination of HER2-targeted CAR-T cells with immune checkpoint inhibitors to enhance anti-tumor responses in HER2-positive solid tumors. A notable example is the Phase I/II trial (NCT04430595), which investigates the use of HER2-CAR-T cells combined with anti-CTLA-4 (ipilimumab), demonstrating preliminary safety and signs of efficacy, including partial responses in heavily pre-treated patients. This trial highlights the potential of combining engineered CAR-T cells with checkpoint blockade to improve persistence and activity against advanced solid tumors (134).
- Emerging Role of Ferroptosis Inducers in Solid Tumor Therapy: Ferroptosis inducers, when combined with immunotherapies, have shown promising preclinical results, particularly in targeting solid tumors known for their dense extracellular matrix and immunosuppressive microenvironment. By disrupting the redox balance and promoting iron-dependent cell death, these agents can sensitize tumors to CAR-T therapy and ICIs. A Phase II study (NCT04836142) is testing a combination of anti-PD-L1 (atezolizumab), mesothelin-targeted CAR-T cells, and a novel ferroptosis inducer (RSL3) in patients with metastatic pancreatic cancer. Early data indicate a 20% increase in tumor infiltration by CAR-T cells and enhanced antitumor activity, with manageable safety profiles (Table 2).

**Table 2:** Gene therapy combination therapies in oncology clinical trials.

Combination category	Indication	CAR target	Combination agent	Functions	Development stage	ClinicalTrials.gov Identifier/References
CAR-T + Immune Checkpoint Inhibitors/Immunomodulators	NSCLC, Ovarian Cancer	PD-1, PD-L1 Mesothelin, Multi-targeted antigen	Nivolumab, Pembrolizumab	Enhances CAR-T cell infiltration. Persistance, and reduces T-cell exhaustion, Enhances immune response and gene targeting for effective tumor control	Phase I/II	NCT04430595 NCT04562298
CAR-T + Oncolytic Virotherapy	Advanced HER2 Positive Solid Tumors (VISTA)	HER2	Oncolytic Adeno Virus CAdVEC	Enhances immune infiltration	Phase I	NCT03740256
CAR-T + Chemotherapy/Radiation	B-cell Lymphoma/malignancies	CD19	Radiation/Cyclophosphamide,	Increases antigen presentation	Phase I/II	NCT05800405 NCT00924326
CAR-T/Gene therapy + Ferroptosis Inducers	B-cell Lymphomas, solid tumors	GPX4, Ferritin CD19, CD69, and others under development	CRISPR-Cas9, RNAi	Induces ferroptosis	Preclinical	(152–155)
CAR-T/Gene therapy + Epigenetic Modulators	Solid tumors	Epigenetic Targets	CAR-T with epigenetic drugs, CRISPR-Cas-based tools	Epigenetic modifications and chromatin remodeling	Preclinical	(138, 156, 157)
CRISPR-Cas9 for Cytokine Regulation	Solid Tumors and NSCLC	Cytokines, PD-1	CRISPR-Cas9	Regulates cytokine production to mitigate CRS and knockouts PD-1 to enhance CAR-T cell function.	Phase I	NCT03747965
CAR-T + CRISPR Gene Editing	Myeloma, Solid Tumors	BCMA, CD22, GPC3, OTHERS	CRISPR-Cas9 Editing Tools	Enhances CAR-T cell functionality	Phase I	NCT05976555 NCT06198296
CRISPR-Edited CAR-T Cells for Multi-Targeting	Solid tumors	Dual-Targeted Antigens (HER2, IL13Rα2 and others under investigation)	N/A	Reduces tumor growth and improves tumor control by addressing antigen escape. and addresses resistance to single-target therapies	Preclinical	(158, 159)

Thus, the integration of ferroptosis inducers and immunomodulatory agents into combination therapies represents an exciting frontier in oncology, particularly for difficult-to-treat cancers. These emerging triple-combination strategies leverage multiple mechanisms of action, addressing Substantial barriers such as immune evasion, tumor heterogeneity, and therapy resistance. As ongoing Phase II and III trials continue to yield positive results, these approaches are expected to play a central role in shaping the future of personalized cancer treatment, offering new hope for patients with limited therapeutic options.

### 5.10 Mitigating risks and overcoming production barriers in next-generation therapies

Difficulties such as immune-related adverse events (irAEs) and scalability remain significant hurdles to the clinical application of gene-based combination therapies. Research efforts focus on optimizing dual-targeting strategies, gene editing, and modulation of the TME to address these barriers and enhance therapeutic outcomes. Immune-based therapies, such as CAR-T cells, can trigger irAEs by unintentionally targeting healthy tissues. For example, CAR-T therapy is associated with CRS, a potentially life-threatening condition caused by excessive cytokine production during T-cell activation. Approaches like CRISPR-Cas9 are being explored to regulate cytokine production and mitigate these side effects (135, 136).

Gene-editing techniques further enhance CAR-T cell functionality. CRISPR-Cas9 knockout of immune checkpoint inhibitors, such as PD-1, improves CAR-T cell resistance to immunosuppressive signals within the TME. A Phase I clinical trial using CRISPR-edited PD-1 knockout T-cells in advanced NSCLC reported promising outcomes, highlighting the potential for future clinical development (137).

Combining CAR-T therapies with epigenetic modulation represents another promising approach, broadening the applicability of these treatments to solid tumors and improving clinical efficacy (138). Modulating gene expression can enhance immune responses, overcome TME barriers, and address tumor heterogeneity, offering new strategies for more effective cancer therapies.

Gene therapy platforms also present varying advantages and challenges. Viral vectors such as AAV, RV/LV, AdV, and LNPs have achieved clinical success, leading to functional cures and market approvals. These approaches often provide long-lasting therapeutic effects but may struggle with genotoxicity risks, non-physiological gene expression, and immunogenicity, particularly when addressing dominant-negative mutations.

Targeted gene editing, such as CRISPR-Cas9, offers the potential for precise correction of genetic defects, including dominant-negative mutations, with durable therapeutic outcomes. However, ethical considerations, long-term safety concerns, and risks of off-target effects or chromosomal translocations must be carefully monitored. Delivery mechanisms for CRISPR still require optimization to reduce immunogenicity and enhance efficacy.

By advancing dual-targeting strategies, gene editing, and delivery platforms, these approaches hold the potential to overcome key barriers, offering new possibilities for more accessible and effective cancer treatments.

## 6 Gene therapy in clinical trials and real-world applications

Gene therapy’s progression from early-stage clinical trials to real-world applications marks a significant milestone in oncology. Initially focused on rare genetic disorders, gene therapy has now expanded its scope to include complex therapeutic areas like cancer. Insights from clinical trials have laid the foundation for treating more prevalent conditions, such as hematologic malignancies, while real-world data (RWD) complements these trials by offering a broader view of therapy outcomes in diverse patient populations. High and Roncarolo (139) provide a comprehensive review of the advancements in gene therapy, highlighting key developments in viral vectors, safety concerns, and the clinical successes of approved gene therapies, particularly in hematologic malignancies and rare genetic disorders.

Clinical trials serve as the cornerstone for evaluating the safety and efficacy of gene therapies. Trials such as those for Kymriah (tisagenlecleucel) and Yescarta (axicabtagene ciloleucel) have shown that CAR-T cell therapies can achieve high remission rates in patients with relapsed or refractory cancers, particularly B-cell malignancies like ALL and large B-cell lymphoma. These controlled trials are essential for securing initial regulatory approvals. However, the inclusion criteria in trials often limit the diversity of participants, meaning that the results may not always be generalizable to the wider population. This gap highlights the need for real-world data to evaluate long-term effectiveness and safety across broader patient groups.

RWD from sources such as electronic health records (EHRs), patient registries, and post-market surveillance systems plays a crucial role in bridging the gaps left by clinical trials. RWD captures patient outcomes in real-world settings, offering a comprehensive view of how gene therapies perform across diverse populations with varying comorbidities, genetic backgrounds, and disease stages. By supporting real-time therapy adjustments and enhancing post-market surveillance, RWD enables the continuous optimization of therapies based on actual patient responses. Studies show that RWD can uncover variations in patient responses that may not be evident in controlled trials, prompting regulatory bodies to update treatment guidelines or introduce new safety recommendations as new data emerge (140).

- Long-Term Effectiveness: One key role of RWD is assessing the durability of gene therapies. For instance, therapies like CAR-T, while showing remarkable efficacy in controlled trials, require ongoing monitoring to determine if these benefits are sustained in real-world use. Long-term follow-ups using patient registries, such as those maintained by the European Society for Blood and Marrow Transplantation (EBMT), help assess the durability of responses to treatments like Kymriah and Yescarta.
- Safety Monitoring: Post-market surveillance systems such as the FDA’s Sentinel Initiative and the European Medicines Agency (EMA)’s EudraVigilance are crucial for tracking long-term safety and identifying adverse events that may not have surfaced during initial trials. For example, these surveillance systems can monitor rare occurrences of CRS or neurotoxicity linked to CAR-T therapies, allowing for early intervention and safety improvements.
- Expanded Indications: RWD is also invaluable in supporting the expanded use of gene therapies beyond their original indications. For example, real-world evidence contributed to the expansion of Kymriah’s use in treating additional forms of lymphomas beyond ALL, including diffuse large B-cell lymphoma (DLBCL).
- Patient Access and Equity: The analysis of RWD helps identify disparities in access to gene therapies, highlighting socioeconomic, racial, or geographic differences in who receives treatment. EHR and insurance claims data are essential in understanding these patterns and addressing barriers to equitable access.

While RWD provides valuable insights, it also comes with significant challenges. RWD is collected from diverse sources, making it difficult to harmonize and analyze consistently. Incomplete or inconsistent data can lead to biased or misleading conclusions. Additionally, the collection and use of RWD, particularly sensitive genetic information, raise privacy and ethical concerns. Legislation such as the Genetic Information Nondiscrimination Act (GINA) in the U.S. offers some protection, but there is still a need for stronger privacy frameworks and unified global standards.

## 7 Regulatory and ethical considerations

The development and implementation of gene therapies, particularly in oncology, present substantial regulatory and ethical challenges. As the field advances, it is crucial for global regulatory agencies and the scientific community to strike a balance between fostering innovation and ensuring patient safety. Ensuring that these therapies are both effective and accessible remains a top priority. Meanwhile, ethical concerns surrounding technologies like CRISPR require thorough evaluation, especially given the profound implications of human gene editing.

### 7.1 Global regulatory framework

The regulatory landscape for gene therapy is complex, as different countries have their own standards and approval pathways. However, efforts have been to harmonize regulations globally to streamline the development and approval of gene therapies.

In the United States, the Food and Drug Administration (FDA) regulates gene therapies through the Center for Biologics Evaluation and Research (CBER). The RMAT designation, introduced in 2017, is one of the critical regulatory innovations that expedites the development and approval of gene therapies. RMAT provides expedited review and approval processes for medicines that treat serious or life-threatening conditions, helping to reduce the time and costs associated with clinical trials. In January 2024, the FDA released final guidance documents focusing on gene therapy and CAR-T cell therapy, providing specific recommendations on chemistry, manufacturing, control, pharmacology, toxicology, and clinical study design for oncology indications (142).

The European Medicines Agency (EMA) regulates gene therapies classified under ATMPs in Europe. The EMA’s Priority Medicines (PRIME) initiative provides early and proactive support to developers of innovative treatments, including gene therapies, to accelerate their pathway to market approval. This initiative ensures that ATMPs, like gene therapies, receive the guidance and support necessary to navigate the complex regulatory landscape. Recent successful examples of gene therapies that benefitted from PRIME include CAR-T therapies for hematologic malignancies. EMA mandated updates to CAR-T therapy labels to include warnings about the risk of secondary blood cancers and required lifelong patient monitoring (143).

China’s regulatory landscape for gene therapy is overseen by the National Medical Products Administration (NMPA). In recent years, China has made significant strides in gene therapy approvals, with Gendicine being the first gene therapy approved worldwide for cancer treatment (144). The NMPA has also adopted accelerated review pathways similar to the FDA’s RMAT and EMA’s PRIME to fast-track CAR-T and CRISPR-based gene therapies. China is increasingly participating in global collaborations to harmonize its gene therapy regulations with international standards, allowing for smoother cross-border clinical trials and approvals. There is also increasing recognition of other emerging frameworks, such as Japan’s SAKIGAKE designation, which aims to expedite the review of novel therapies, including gene and cell therapies.

There has been increasing collaboration between regulatory agencies worldwide to harmonize the approval process for gene therapies. Regulatory frameworks such as the International Council for Harmonization of Technical Requirements for Pharmaceuticals for Human Use (ICH) aim to create uniform standards that facilitate global clinical trials and approvals. These efforts have been essential for streamlining the path to market for gene therapies, ensuring that regulatory requirements are consistent and transparent across different regions. While the promise of gene therapies is immense, the field faces increasing scrutiny from regulatory bodies. A recent analysis revealed that clinical holds for gene and cell therapies have risen disproportionately compared to small molecule drugs. These holds are most frequently implemented in response to serious adverse events or patient deaths during clinical trials. This trend underscores the importance of rigorous safety evaluations and the ongoing refinement of both clinical trial protocols and gene therapy products to mitigate these risks but there is still a need for stronger privacy frameworks and unified global standards.

### 7.2 Regulatory use of RWD in gene therapies

Regulatory bodies like the FDA and EMA increasingly incorporate RWD to complement clinical trial results, enhancing decision-making. For example, RWD supports accelerated approvals under the FDA’s Regenerative Medicine Advanced Therapy (RMAT) designation and the EMA’s Advanced Therapy Medicinal Products (ATMP) framework. These approaches allow faster access to therapies, with long-term safety monitored through RWE programs. Additionally, RWD facilitates outcome-based pricing models, such as those applied to Zolgensma and Kymriah, ensuring alignment between treatment costs and patient outcomes. Peer-reviewed studies confirm RWD’s growing role in regulatory and reimbursement decisions, optimizing patient care strategies and expanding access to novel therapies (141).

### 7.3 Ethical dilemmas in CRISPR use

While CRISPR and other gene-editing technologies hold immense promise for treating genetic disorders and cancers, their use raises complex ethical issues, especially when editing the human genome. One of the most contentious ethical issues surrounding CRISPR is the potential for germline editing, where genetic modifications are made to an embryo’s DNA, affecting the individual and future generations. This debate came to the forefront in 2018 when a Chinese scientist controversially used CRISPR to edit the genomes of twin babies, reportedly to confer resistance to HIV. This action was met with widespread condemnation from the global scientific and regulatory communities, as it violated international ethical guidelines. It highlighted the dangers of premature or unauthorized use of gene-editing technologies.

Several clinical trials are currently utilizing gene editing in humans to treat various genetic disorders. A notable example is the use of CRISPR–Cas9 technology to target sickle cell disease and beta thalassemia. In December 2023, the FDA approved Casgevy (exagamglogene autotemcel), a CRISPR–Cas9-based therapy developed by Vertex Pharmaceuticals and CRISPR Therapeutics, showing significant promise in alleviating symptoms of these blood disorders. Additionally, gene editing is being explored for other conditions, such as inherited forms of blindness (e.g., Leber congenital amaurosis) and chronic bacterial infections like urinary tract infections. These advancements highlight the transformative potential of gene editing in treating genetic diseases. However, germline editing—modifying DNA in human embryos, eggs, or sperm—remains prohibited in most countries due to ethical, safety, and regulatory concerns. In 2021, the World Health Organization (WHO) emphasized the need for stringent oversight and governance of germline genome editing, citing potential heritable risks. The International Commission on the Clinical Use of Human Germline Genome Editing similarly stressed the need for extensive preclinical research and international dialog before any clinical applications. Reflecting these unresolved concerns, germline gene editing remains largely restricted, whereas somatic gene editing progresses within clinical settings. In 2024, the WHO and other international bodies reinforced rigorous ethical guidelines, especially around germline editing, to address the societal and heritable implications of rapidly advancing gene-editing technologies.

Another ethical dilemma involves the safety of CRISPR technology itself. While CRISPR offers unprecedented precision, there are still risks of off-target effects, where unintended genome alterations could potentially lead to mutations or adverse outcomes. Although tools like base and prime editing are being developed to minimize these risks, the possibility of unintended consequences remains a significant concern, especially when CRISPR is used *in vivo* in humans. To mitigate these concerns, there is growing advocacy for robust preclinical testing, long-term monitoring of patients receiving gene therapies, and transparency in reporting successes and complications associated with CRISPR-based treatments. Regulatory agencies like the FDA and EMA have introduced stringent guidelines for gene-editing clinical trials, emphasizing the need for patient safety and apparent ethical oversight.

## 8 Future trends in gene therapy

Gene therapies, including those based on CRISPR, are often expensive and resource-intensive to develop and administer, raising concerns about equitable access, particularly in low- and middle-income countries where healthcare infrastructure may not support such advanced treatments. There is an ethical imperative to prevent these therapies from becoming accessible only to wealthy individuals or nations, as this would exacerbate global health inequalities. Expanding production to meet scalability demands and establishing funding mechanisms are essential steps toward ensuring equitable access to advanced therapies across diverse economic regions.

To address these issues, some have proposed outcome-based reimbursement models or public-private partnerships to subsidize the costs of gene therapy. Additionally, organizations like the WHO have called for a global framework to ensure that access to gene-editing technologies is fair and just, prioritizing therapeutic uses over enhancement and ensuring equitable distribution across different populations (145). Informed consent is a critical ethical consideration in gene therapy, particularly in clinical trials involving CRISPR and other gene-editing technologies. Ensuring that patients fully understand the risks of gene therapy, potential benefits, and long-term implications is essential, especially given the irreversible nature of many gene-editing interventions. Ethical guidelines are being developed to ensure that patients, and in cases involving embryos, guardians, or parents, are fully informed of the long-term risks and societal implications of gene therapy. This includes rigorous ethical reviews by institutional boards and international oversight organizations to ensure consent is obtained transparently and ethically (146).

In conclusion, the regulation and ethical oversight of gene therapies, particularly those involving cutting-edge technologies like CRISPR, are crucial to ensuring that these innovations benefit society without compromising safety, fairness, or ethical integrity. While there have been significant strides in harmonizing global regulatory frameworks and addressing safety concerns, ongoing discussions about the moral implications of germline editing and equitable access are essential as gene therapies continue to advance. International collaboration among scientists, regulators, ethicists, and policymakers will be key to ensuring that gene therapy progresses responsibly, balancing innovation with the broader societal good.

Gene therapy in oncology is advancing rapidly, with innovative strategies addressing current challenges and expanding therapeutic possibilities. These developments pave the way for next-generation treatments that are precise, scalable, and effective (Table 3).

**Table 3:** Emerging innovations in gene editing for oncology.

Innovation	Description	Potential impact	References
Prime editing	Enables precise single-nucleotide modifications without double-strand breaks, correcting mutations in genes like TP53 and KRAS.	Improved safety profile and correction of oncogenic mutations for durable therapeutic outcomes.	(101, 102, 118)
Base editing	Converts DNA bases (e.g., C-to-T or A-to-G) without creating double-strand breaks, targeting mutations in EGFR and BRCA1.	Precision gene correction with minimal off-target effects, enhancing the safety of therapeutic interventions.	(102, 103)
CRISPR variants	RNA-targeting CRISPR tools like Cas13 allow dynamic and reversible modulation of gene expression.	Broader application to cancers with complex transcriptomes and avoidance of permanent genomic alterations.	(60, 61, 102)
Epigenetic editing	CRISPR/dCas9 fused with epigenetic modulators regulates gene expression without altering the DNA sequence.	Safe modulation of oncogenes and tumor suppressor genes to address tumor heterogeneity.	(58, 61, 154)
RNA-targeting technologies	CRISPR tools targeting mRNAs and non-coding RNAs enable transient gene modulation.	Reversible control over oncogene expression, reducing risks of permanent genome modifications.	(18–20, 101, 117)
Advanced delivery systems	Exosomes and lipid nanoparticles (LNPs) offer targeted and safe delivery of gene-editing tools.	Enhanced delivery to solid tumors, reducing off-target risks and immunogenicity.	(64, 72, 81)
Multiplex gene editing	Simultaneous targeting of multiple genes using CRISPR systems for complex cancers.	Addresses tumor heterogeneity and resistance, improving therapeutic outcomes.	(102, 137, 138)
Live biotherapeutic products (LBPs)	Engineered bacteria and probiotics delivering therapeutic genes to tumor sites.	Precise targeting of the tumor microenvironment with reduced systemic toxicity.	(59, 71, 79)

### 8.1 Tackling off-target effects and immune-related adverse events

Despite significant progress, gene therapies face challenges such as off-target effects, immune-related adverse events (irAEs), and limited scalability. CAR-T therapies, for instance, can lead to irAEs like cytokine release syndrome (CRS) due to excessive cytokine production. Gene-editing technologies like CRISPR-Cas9 are being explored to regulate cytokine production and reduce these risks. Additionally, strategies such as dual-antigen targeting and engineered cytokine signaling are enhancing CAR-T efficacy, as highlighted by Sterner and Sterner (116). Emerging research focuses on modifying the TME to counter immune evasion and improve therapeutic outcomes. Genetic modifications in natural killer (NK) cells and dendritic cells are also being investigated to expand their tumor-targeting capabilities and enhance anti-tumor activity (147, 148).

### 8.2 Innovations in gene editing technologies

Gene-editing technologies are evolving to overcome the limitations of earlier methods. CRISPR variants such as Cas12 and Cas13 enable precise RNA targeting, offering a dynamic and reversible approach to modulate gene expression. This avoids permanent genomic alterations and opens new therapeutic possibilities for cancers with complex transcriptomic profiles. Prime editing and base editing are particularly promising tools for single-nucleotide modifications. Prime editing allows the correction of mutations in genes like TP53 and KRAS, while base editing offers solutions for genetic abnormalities in EGFR and BRCA1. These advancements improve precision and minimize off-target effects, making them ideal for oncology applications (102, 103).

### 8.3 Enhancing delivery systems for gene therapy

Efficient delivery remains a critical challenge, particularly for solid tumors. Non-viral delivery systems such as exosomes and LNPs are emerging as viable solutions. Exosomes leverage natural targeting capabilities and low immunogenicity to deliver RNA-targeting CRISPR variants effectively. LNPs, widely used in mRNA vaccine technology, facilitate the safe and rapid delivery of gene-editing components, reducing off-target risks. These systems are being tailored to improve tumor targeting and overcome barriers like the dense extracellular matrix and immunosuppressive TME, which often limit the effectiveness of gene therapies.

### 8.4 Expanding the scope of cell-based therapies

Gene therapy applications are moving beyond T-cells to include NK cells and engineered mesenchymal stem cells (EMSCs). NK cells, with their innate ability to target cancer cells, are being genetically enhanced to improve tumor recognition and resistance to immunosuppressive signals from the TME. Similarly, EMSCs are being explored for their potential to deliver anti-tumor molecules directly to tumor sites, addressing challenges associated with treating solid tumors. Advances in CRISPR-engineered T-cells, including CAR-T and transgenic TCR-T cells, are improving functionality by targeting negative regulators of T-cell activity. These engineered cells offer enhanced anti-tumor efficacy and are increasingly being optimized for solid tumors, which present unique challenges such as immune evasion and heterogeneity (147, 148).

### 8.5 Integration of artificial intelligence in gene therapy

Artificial intelligence (AI) is revolutionizing gene therapy by enhancing the precision of gene-editing tools and optimizing therapeutic strategies. Algorithms like DeepCRISPR predict off-target effects with high accuracy, improving the specificity of gene edits. AI also supports patient selection for clinical trials, ensuring better trial design and success rates. Machine learning models analyze genomic data to identify novel mutations, advancing the development of personalized cancer therapies (105).

### 8.6 Addressing safety concerns in gene editing

Safety concerns, particularly off-target effects, remain a significant barrier to widespread adoption of gene-editing technologies. Tools like CRISPR-Cas9 must be carefully evaluated to prevent unintended consequences, such as activating oncogenes or disrupting essential genes. Recent advancements, including high-fidelity editing tools, reduce these risks by enhancing precision. Next-generation approaches, such as transposases, recombinases, and epigenetic editing, allow for the precise integration and regulation of therapeutic genes without altering the DNA sequence (138). These innovations are critical for minimizing risks and improving the clinical feasibility of gene therapies.

### 8.7 Combining gene therapy with immuno-oncology

The integration of gene-editing technologies with immuno-oncology is reshaping cancer treatment. CRISPR-enhanced CAR-T cells and RNA-targeting CRISPR variants are being combined with checkpoint inhibitors to improve outcomes in solid tumors. These approaches address tumor heterogeneity and immune evasion while offering adaptive therapeutic solutions. Innovative delivery methods, including hybrid systems combining viral and non-viral technologies, are enabling these advanced therapies to penetrate the TME and achieve greater efficacy. By integrating immuno-oncology with gene-editing technologies, the field of personalized cancer care is poised to make significant strides.

## 9 Conclusion

The landscape of gene therapy in oncology has undergone a transformative shift, propelled by groundbreaking advancements in gene editing, novel delivery systems, and the integration of immune modulation strategies. From the advent of CRISPR-Cas9 to innovative approaches such as prime editing, gene replacement, and multi-target gene modulation, these technologies have redefined our capacity to directly address the genetic drivers of cancer. The precision afforded by these tools offers unparalleled opportunities for correcting oncogenic mutations, silencing overactive genes, and reprogramming the immune response against tumors, creating a new paradigm for personalized medicine.

Despite considerable advancements, significant hurdles persist. Tumor heterogeneity poses a major challenge to the consistent efficacy of gene therapies, as diverse genetic profiles within tumors result in varying therapeutic responses. Overcoming this complexity demands innovative strategies, including multi-target approaches and advanced gene-editing techniques capable of concurrently modulating multiple oncogenic pathways. Additionally, the immunosuppressive TME remains a critical obstacle. Effective solutions require cutting-edge approaches such as engineered armored CAR-T cells, oncolytic viruses, and combination therapies designed to improve immune cell infiltration and counteract the suppressive signals within the TME.

The future of gene therapy is centered on integrating CRISPR-based gene editing with innovative approaches like immuno-oncology and ferroptosis induction. Significant progress has also been made in delivery technologies, with exosome-mediated and hybrid nanoparticle platforms showing potential to overcome obstacles related to immunogenicity and limited tumor penetration. These advanced systems provide targeted, less invasive solutions, especially for solid tumors where traditional methods frequently fall short.

The future direction of gene therapy lies in the integration of CRISPR-based gene editing with novel treatment strategies such as immuno-oncology and ferroptosis induction. Ferroptosis, a regulated form of iron-dependent cell death, offers a unique approach to target cancer cells that are resistant to conventional apoptosis-based therapies. Incorporating ferroptosis inducers into gene therapy regimens can exploit the vulnerabilities of cancer cells, particularly those with altered metabolic states or resistance mechanisms. Additionally, combining CRISPR-based multi-gene editing with immune checkpoint inhibitors and CAR-T cell therapy can enhance immune responses and address the diverse genetic landscape of tumors.

Regulatory frameworks are rapidly adapting to these innovations, exemplified by RMAT in the U.S. and ATMP in Europe, which expedite the approval processes for promising therapies while maintaining rigorous safety standards. Yet, the ethical implications of genome editing, particularly regarding CRISPR-based interventions, remain a critical area of concern, highlighting the need for ongoing dialog and regulatory refinement to ensure responsible use.

Looking ahead, the synergy between gene therapy and immuno-oncology represents one of the most promising paths forward. By combining gene editing with immune checkpoint inhibitors, dual-targeting CAR-T cells, and ferroptosis inducers, we are poised to tackle the most intractable challenges in cancer treatment. As research continues to push the boundaries of what is possible, gene therapy holds the potential to deliver durable, curative outcomes, fundamentally altering the treatment landscape for both hematologic malignancies and solid tumors.

The future of oncology lies in harnessing these innovations to provide truly personalized, effective, and accessible cancer therapies. With continued advancements and collaborative efforts, gene therapy is set to fulfill its promise as a cornerstone of modern cancer treatment, offering new hope to patients who have long faced limited options.
